# A clinical trial report of autologous bone marrow-derived mesenchymal stem cell transplantation in patients with spinal cord injury

**DOI:** 10.3892/etm.2013.1083

**Published:** 2013-04-29

**Authors:** PU-CHA JIANG, WEN-PING XIONG, GE WANG, CHAO MA, WEI-QI YAO, STEVEN F. KENDELL, BRIAN M. MEHLING, XIAN-HOU YUAN, DONG-CHENG WU

**Affiliations:** 1 Department of Neurosurgery, Zhongnan Hospital, Wuhan University, Hubei 430071, P.R. China;; 2 Biochemistry Institute, Wuhan University, Hubei 430071, P.R. China;; 3 Department of Stem Cells, Wuhan Hongqiao Brain Hospital, Wuhan, Hubei 430071, P.R. China;; 4 Blue Horizon International, LLC, New York, NY 10014, USA;; 5 Department of Neurosurgery, Zhongnan Hospital of Wuhan University, Wuhan, Hubei 430071, P.R. China

**Keywords:** spinal cord injury, autologous bone marrow-derived mesenchymal stem cells, human bone marrow-derived mesenchymal stem cells, clinical trial

## Abstract

Spinal cord injury (SCI) is a severe neurological disease. An effective strategy for the treatment of SCI is urgently required. Stem cell transplantation has emerged as a viable therapeutic option with great potential for restoring neurological function lost following SCI. From 2009 to 2010, a total of 20 SCI patients were enrolled in a clinical trial by Wuhan Hongqiao Brain Hospital; all patients completed and signed informed consent prior to autologous bone marrow-derived mesenchymal stem cell transplantation. Analysis of subsequent treatment results indicated significant improvements in sensory, motor and autonomic nerve function as assessed by the American Spinal Injury Association’s impairment scale. Thirty days after transplantation, a total of 15 patients (75%) demonstrated improvement, including four of the eight patients (50%) with grade A SCI, three of the four patients (75%) with grade B injury and all eight patients (100%) with grade C injury. The most common adverse events, fever and headache, disappeared within 24–48 h without treatment.

## Introduction

The repercussions of spinal cord injury (SCI), regardless of origin, are often severe and include loss of bowel and bladder faculties, impairments in sensory and motor function and intractable pain. Despite current treatment strategies, including surgical decompression and fixation, the injection of neurotropic factors, anti-inflammatory medications and physical rehabilitation, satisfactory therapeutic effects remain elusive. Although a steadily accruing body of evidence points to the central nervous system possessing a means for self-repair, this capacity appears quite limited as a sole clinical approach. The complex pathology of SCI may be divided into primary and secondary injury. The primary injury is characteristically induced by mechanical damage and resultant hemorrhage. Myriad factors contributing to secondary injury include: excitatory amino acid toxicity, oxidative damage, inflammation and autoimmune response. These combined injury mechanisms, leading to glial and neuronal cell death, demyelinization and axonal degeneration, are manifested as a severe impairment in neurological function (1).

Stem cell transplantation has emerged as a possible alternative therapy for refractory nervous system conditions, including SCI (2,3). Approved by the Food and Drug Administration (FDA) of the United States of America and the State Food and Drug Administration (SFDA) in China, a limited number of clinical trials concerning stem cell transplantation have, consequently, been published (4–6). Human bone marrow-derived mesenchymal stem cells (HBMSCs), identified alongside hematopoietic stem cells and possessing tremendous capacity for self-renewal and differentiation, are a type of adult stem cell that have demonstrated positive effects in the treatment of SCI (7–9). In the current study, we report on the safety and therapeutic efficacy of autologous HBMSC transplantation in 20 SCI patients treated at Wuhan Hongqiao Brain Hospital, China.

## Patients and methods

### 

#### Cases

A total of 20 SCI patients were enrolled in our study at the Wuhan Hongqiao Brain Hospital from 2009 to 2010. There were 13 male patients (65%) and 7 female patients (35%) aged 9–72 years, with an average age of 41.1 years. The maximum duration since the time of SCI was 10 years with a minimum time of 3 months (Table I). According to the American Spinal Injury Association’s classification of SCI (ASIA impairment scale) there were 8 cases (6 males and 2 females) of grade A, 4 cases (1 male and 3 females) of grade B and 8 cases (6 males and 2 females) of grade C. Among all SCI patients, there were 4 cases involving the cervical region, 11 cases involving the thoracic region and 5 cases involving the lumbosacral region. When divided into the etiology of SCI, there were 5 cases caused by car accidents, 7 cases caused by violence, 2 cases caused by a fall and 6 cases not caused by trauma. This study was conducted in accordance with the Declaration of Helsinki and with approval from the Ethics Committee of Wuhan Hongqiao Brain Hospital. Written informed consent was obtained from all participants.

### Transplantation methods

#### Preparation of HBMSCs

Under aseptic conditions, a puncture surgery was conducted, isolating 10 ml bone marrow from the upper edge of the patient’s iliac bone. The collected bone marrow was immediately anticoagulated by heparin and sent to the laboratory. HBMSCs were isolated and cultured in a laboratory following good manufacturing practice (GMP). Cultured cells were passaged weekly and, after 3 weeks of passage, when 1×10^8^ cells were produced, the cells were subsequently stored in liquid nitrogen until required.

#### Cell transplantation

One hour prior to transplantation, the cells were resuspended in 5 ml saline. The patients received transplanted stem cells via lumbar puncture or by computed tomography (CT)-guided injection that directly targeted the lesion sites. A total of 5 ml (1×10^8^) BMSCs were injected into each SCI patient.

#### Neurological grading

Neurological grading was performed using the ASIA impairment scale as follows: Grade A, complete: no motor or sensory function is preserved in the sacral segments S4-S5; grade B, incomplete: sensory but not motor function is preserved below the neurological level and includes the sacral segments S4-S5; grade C, incomplete: motor function is preserved below the neurological level and more than half of key muscles below the neurological level have a muscle grade <3; grade D, incomplete: motor function is preserved below the neurological level and at least half of the key muscles below the neurological level have a muscle grade of ≥3; and grade E, normal: motor and sensory function is normal.

All the patients were assessed for ASIA rating on the day prior to transplantation, as well as at regular intervals following treatment.

#### Muscle tone, abnormal motion and paresthesia

The Ashworth scale of muscle spasticity is considered a valid measure of increasing muscle tone but not of reduced muscle tone. SCI, however, may lead to either an increase or reduction in muscle tone. Consequently, in the present clinical trial, the physical characteristics of muscle changes were recorded instead of a score according to the Ashworth scale. Patients’ description of abnormal motion and sensation, including zonesthesia, numbness and hyperesthesia were also reported.

#### Urinary and bowel function

Incontinence is a common issue for SCI patients. Assessments of bowel and bladder function are integral components of the Barthel activities of daily living (ADL) index. In order to better qualify the Barthel ADL index measures, however, these measures were subdivided. Bladder dysfunction was divided into no automatic micturition, incontinence, difficulty in urination, poor urine control and dribbling urine. Bowel dysfunction was divided into constipation, fecal incontinence and dry stools.

#### Pain

Pain, a common and typically severe sequelae, has tremendous impact on the daily life of SCI patients. Although the pathophysiology of pain has yet to be fully defined in SCI patients, its measure, considered integral, is classified as a separate therapeutic index. A subjective description of daily emotional state was also recorded and used for assessment post-transplantation.

#### Erectile dysfunction (ED)

An erection may be induced physically, by reflex or psychologically (mental erection). Previous studies have shown that 60–70% of SCI patients desired sexual relations (10); however, in China, patients remained reluctant to discuss their sexual health issues due to cultural traditions. As a consequence of this cultural taboo, a full assessment of erectile issues was not completed; however, the information from the limited number of individual SCI patients willing to discuss this subject was assessed.

## Results

### 

#### Neurological grading by ASIA impairment scale

Our analysis of the 20 SCI study patients receiving autologous HBMSC transplantation was notable for all patients exhibiting improvement in sensory and motor function. Of the 20 SCI study patients, 15 individuals (75%) improved by one or two grades as measured by the ASIA impairment scale, with 14 (93.3%) of those 15 patients improving by one grade and 1 (6.7%) of the 15 patients improving by two grades (Table II).

As described in Table II, a lower ASIA rating prior to transplantation resulted in a slower recovery. Our data suggested that autologous HBMSC transplantation had a greater therapeutic effect in moderately injured patients than in severely injured patients. There were 14 (93.3%) patients whose condition increased by one grade and 1 patient (6.7%) whose condition increased by two grades; however, no patient’s condition increased by three grades.

The prognosis of SCI patients appears to be dependent on the stage of SCI, the cause(s) of SCI and patient age. Considering the multiple disparate characteristics of our study patients, we compared the therapeutic effects of autologous HBMSC transplantation on SCI patients according to the variables in Table III.

Our clinical data, although lacking a randomized control group, was based on the statistical analysis of our SCI cohort. The data was analyzed by χ^2^ test, with α set at 0.05. Limitations in data set size, however, precluded statistical significance being reached for time of injury, cause of injury, age and gender, as they relate to the efficacy of stem cell transplantation for SCI.

#### Assessment of urinary and bowel function

Of the 10 individuals (50% of the total SCI study subjects) suffering from urinary dysfunction, pre-stem cell treatment classification included: 1 patient with no automatic micturition, 4 patients with incontinence, 3 patients with difficulty in urination, 1 patient with poor urine control and 1 patient with dribbling urine. Patient recovery information is shown in Table IV. No automatic micturition is defined as urinary retention requiring catheterization. Difficulty in urination refers to laborious, non-smooth urine production. Of the ten SCI individuals suffering from urinary dysfunction, 80% (8/10) experienced post-stem cell transplantation improvement in urinary function to varying degrees.

Of the 12 individuals (60% of the total SCI study subjects) suffering from bowel dysfunction, pre-stem cell treatment classification included: 5 patients with constipation, 5 patients with fecal incontinence and 2 patients with dry stools. Patient recovery information is listed in Table V. Of the 12 individuals suffering from bowel dysfunction, 9 cases (60%) experienced post-stem cell transplantation improvement in bowel function to varying degrees.

#### Changes of muscle tone, abnormal motion and sensation

Prior to stem cell transplantation, abnormal muscle tone was measured in 13 SCI patients, with 6 patients experiencing reduced muscle tone and 7 patients experiencing increased muscle tone. Patient recovery information is listed in Table VI.

As normal muscle tone is the basis for coordinated human motor function, perturbations in natural muscle tone manifest as problems in daily functioning. As previously noted, the Ashworth scale of muscle spasticity is a common standard used for the assessment of increasing muscle tone. Table VI shows that HBMSC transplantation had a greater effect on qualitatively improving increased rather than reduced muscle tone issues in SCI patients.

Abnormal, non-autonomic movement occurred in the two lower extremities in 1 of the 20 patients following SCI. Six patients complained of abnormal sensations, including zonesthesia, electric shock-like sensation, numbness and hyperesthesia (Table VII).

Although improvement in abnormal motion was not noted, all the patients with zonesthesia improved following cell transplantation, with certain patients experiencing complete resolution of this abnormal sensation.

#### Assessment of pain and ED

Neuralgia, particularly in the extremities, is a well-documented phenomenon in SCI patients (11). Four of the patients in the current clinical trial reported neuralgia with three patients characterizing extremity pain and one patient with headache; this neuralgia, as noted, was greatly improved following HBMSC transplantation (Table VIII).

A previous study reported that ∼25% of SCI patients experience ED (12). As is customary for traditional cultures, discussion of sexuality is considered a taboo. For this reason only two of our male SCI patients were willing to discuss erectile issues; of the two male SCI patients, one experienced improvement in penile sensitivity and tumescence following HBMSC transplantation.

## Discussion

A series of animal experiments (13–17) and clinical trials (18–23) have previously demonstrated that stem cells have beneficial effects for SCI. In the current clinical trial we report on the use of autologous HBMSC transplantation in patients with SCI. Our study demonstrates that multiple sequelae associated with SCI, including sensory and motor dysfunction, abnormal muscle tone, urinary and bowel functional disorders, insufferable pain and ED, may improve significantly following stem cell transplantation.

A study by Yoon *et al* using HBMSC transplantation for the treatment of SCI (18) demonstrated that 29.5% of patients in the acute stage (<2 weeks) experienced an improvement in ASIA impairment rating from grade A to either B or C. Additionally, 33.3% of patients in the subacute stage (2–8 weeks) experienced an improvement in ASIA impairment rating from grade A to either B or C; while no improvement in ASIA impairment scale occurred in the chronic (>8 weeks) group. However, in the current study, 75% of SCI patients experienced an improvement in ASIA rating, with the majority of these patients receiving cell transplantation half a year after SCI, with the exception of one patient who was in the subacute (3 month) SCI stage. Cell therapy appears to have been more beneficial for the patients in the current study than for those in the study by Yoon *et al*, as the patients in our group were of three different ASIA grades prior to cell transplantation whereas all patients in the other study were of grade A. In theory, the higher the grade, the greater the improvement; however, our data suggests an inconsistency with this theory. In addition, while our study followed-up patients for one month, Yoon *et al* continued for 10.4 months. A question, hence, arises as to whether the therapeutic effects of stem cell therapy decline over time and, if verified, what the origin of this phenomenon may be. Once these questions are answered, the benefits of stem cell therapy and its applications may be appreciated.

Abnormal muscle tone in SCI patients includes increases and reductions in muscle tone. For SCI patients, lesion location plays an integral role in the subsequent quality of muscle tone. A lesion in the anterior root or posterior funiculus of the spinal cord leads to reduced muscle tone, while a lesion in the pyramidal tracts causes an increase in muscle tone. In our study all SCI patients with increased muscle tone experienced improvement, while only one-third of SCI patients with reduced muscle tone experienced an improvement. Although statistical significance was achieved with regard to this measure (P=0.021), limitations in sample size make it unclear whether stem cell therapy impacts either increased or reduced muscle tone more substantially.

Normal urinary and bowel function make a significant contribution to quality of life. Of the 10 SCI patients (50% of total) reporting urinary dysfunction, 80% experienced improved micturition following stem cell transplantation. In opposition to the study by Kishk *et al* (23), where no patients experienced complete recovery in urinary function, two patients in our study experienced a return to normal function; one with urinary incontinence and the other with dribbling urine prior to stem cell therapy. Of the 12 patients (60% of total) with bowel dysfunction, 75% experienced an improvement in function following stem cell therapy; this result is commensurate with the results from the study by Kishk *et al*. In addition to aiding the functional integrity of autonomic nerves for normal urinary and bowel function, the return of intestinal secretions following stem cell transplantation may have ameliorated a significant factor in the bowel dysfunction in certain patients.

Pain is a common and severe condition associated with SCI. In the survey by Wrigley *et al* (11), two-thirds of SCI patients suffered from pain that was classified based on the level of injury; while one third of patients suffered from pain, which occurred below the level of injury and was more severe in nature and considered more difficult to treat. Methods used to combat the pain included psychotherapy and the use of anxiolytic and antidepressant medications. However, while two patients from the study demonstrated improvement, further research of stem cell therapy is required to determine the impact of this therapeutic modality on pain.

There were few reports of abnormal sensation brought on by SCI in patients. Of the 6 patients endorsing either zonesthesia, electric shock-like sensations, numbness or hyperesthesia in our study, only the symptom of zonesthesia improved with stem cell therapy; the pathophysiology behind this symptom remains unclear.

Due to the limited number of patients and lack of a control group, we were not able to relate the efficiency of stem cell transplantation to age or gender, nor to time or cause of injury. To obtain statistical significance regarding these variables, a larger number of patients and a control group are warranted.

The most common adverse events in the present study were fever and headache, which disappeared after 1–2 days without any treatment. No patients developed severe adverse effects, further demonstrating the safety of autologous HBMSC transplantation. However, due to the lack of a long-term follow-up, the formation of tumors should not be excluded. To completely assess the possible tumorigenic risk, a long-term follow-up study would be prudent.

Although limitations exist, HBMSC transplantation has demonstrated its effectiveness for the treatment of SCI. The majority of our patients clearly benefited from transplantation with notable improvements in sensory, motor and autonomic function. The mechanisms by which stem cells benefit SCI patients, however, are not fully clear. Currently, the mechanisms by which stem cells are believed to repair damaged tissue include the secretion of neurotrophic factors, the ability to re-wrap injured nerve fibers suffering demyelization and the formation of neural circuitry by transplanted cells that are able to differentiate into neurons (24–30). However, despite steadily accruing evidence in support of the therapeutic benefits of stem cell transplantation, universal consensus regarding the mechanisms of action does not yet exist. Additional studies of autologous HBMSC transplantation for the treatment of SCI remain a critical pursuit.

## Figures and Tables

**Table I.** Basic information of patients when admitted to hospital.

Number	Gender	Age (years)	Injury site	Cause of injury	Time of injury	ASIA grade
1	Male	42	T11-12	Acute hemorrhage in the thoracic vertebrae tract	>1 year	A
2	Male	31	C5-7	Car accident	>1 year	A
3	Male	33	L3-4	Gunshot wound	>1 year	C
4	Male	44	T8	Car accident	6 months	B
5	Female	43	C5-7	Heavy blow	>1 year	C
6	Male	36	T12-L4	Spinal cord schistosomiasis	6 months	A
7	Female	39	T4, 5	Hematoma outside the spinal cord	>1 year	B
8	Male	38	C2-6	Trauma	>1 year	C
9	Male	72	L3-4	Nerve sheath tumor with hemorrhage	7 months	C
10	Male	68	Cervical cord	Fall injury	>1 year	C
11	Female	44	L4-5	Trauma	8 months	A
12	Male	56	T8	Spinal cord atrophy	>1 year	C
13	Female	32	L2-3	Trauma	>1 year	B
14	Female	64	T10-12	Car accident	>1 year	A
15	Male	34	T5, T9, T10	Car accident	>1 year	A
16	Male	30	T12	Heavy blow	3 months	A
17	Female	9	T5-7	Myelitis	>1 year	B
18	Male	38	T2-5	Fall injury	>1 year	A
19	Male	17	S3	Car accident	>1 year	C
20	Female	52	T2	Trauma	>1 year	C

ASIA, American Spinal Injury Association.

**Table II.** ASIA rating before and after cell transplantation.

ASIA rating before transplantation	Initial cases (n)	ASIA rating improvement 30 days after transplantation			No. of cases presenting improvement
		0	1	2	
A	8	4	3	1	4 (50%)
B	4	1	3	0	3 (75%)
C	8	0	8	0	8 (100%)
Total	20	5 (5%)	14 (70%)	1 (5%)	15 (75%)

ASIA, American Spinal Injury Association.

**Table III.** Different variables impacting the efficacy of stem cell transplantation.

Factors	Initial cases (n)	Cases improved 30 days after cell transplantation (n)	Improvement rate (%)	P-value
Time since injury				
<1 year	15	12	80.0	NS (0.56)
>1 year	5	3	60.0	
Cause of injury				
Trauma	14	9	64.3	NS (0.26)
Non-trauma	6	6	100	
Site of injury				
Cervical vertebrae	4	3	75.0	NS (0.646)
Thoracic vertebrae	11	9	81.8	
Lumbosacral	5	3	60.0	
Age (years)				
<18	2	2	100	NS (0.67)
18–60	15	11	73.3	
>60	3	2	66.7	
Gender				
Male	13	11	84.6	NS (0.29)
Female	7	4	57.1	

NS, not significant.

**Table IV.** Recovery of urinary function in spinal cord injury (SCI) patients.

Types	Initial cases (n)	Cases improved 30 days after cell transplantation (n)
No automatic micturition	1	1
Incontinence	4	3
Difficulty in urination	3	2
Poor urine control	1	1
Dribbling urine	1	1

**Table V.** Recovery of bowel function in spinal cord injury (SCI) patients.

Types	Initial cases (n)	Cases improved 30 days after cell transplantation (n)
Constipation	5	4
Fecal incontinence	5	3
Dry stools	2	2

**Table VI.** Recovery of abnormal muscle tone in spinal cord injury (SCI) patients.

Muscle tone	Initial cases (n)	Cases improved 30 days after cell transplantation (n)	Improvement rate (%)	P-value
Increase	7	7	100	
Decrease	6	2	33.3	0.021

**Table VII.** Abnormal motion and sensation.

Types	Initial cases (n)	Cases improved 30 days after cell transplantation (n)
Abnormal motion		
Frequent non-autonomic	1	0
Abnormal sensation		
Zonesthesia	3	3
Numbness	1	0
Hyperesthesia	1	0
Electric shock-like	1	0

**Table VIII.** Recovery of pain and erectile dysfunction (ED) in spinal cord injury (SCI) patients.

Types	Initial cases (n)	Cases improved 30 days after cell transplantation (n)
Pain		
Extremities pain	3	3
Headache	1	1
ED	2	1
